# Supplementary material for: Development and Pharmacokinetics of a Novel Acetylsalicylic Acid Dry Powder for Pulmonary Administration
Source: Pharmaceutics. 2022 Dec 15;14(12):2819. doi: 10.3390/pharmaceutics14122819 (PMC9786194; doi:10.3390/pharmaceutics14122819)
Supplement: Supplementary file 1 [file pharmaceutics-14-02819-s001.zip › pharmaceutics-2045948-supplementary.pdf]

Table S1- ANOVA statistics and coefficients for the models obtained for the four responses of the 2^{6-2} fractional factorial design

	YIELD (%W/W)				ASA (%W/W)				FPF (%W/W)				ED (%W/W)			
**Factors**	**Coefficient estimate**	**Mean Square**	**F-value**	**p-value**	**Coefficient estimate**	**Mean Square**	**F-value**	**p-value**	**Coefficient estimate**	**Mean Square**	**F value**	**p-value**	**Coefficient estimate**	**Mean Square**	**F-value**	**p-value**
**Intercept**	70.31	1359.06	46.99	< 0.0001	66.54	10050.03	362.63	< 0.0001	49.40	1419.74	40.09	< 0.0001	79.29	303.02	4.81	0.0142
**A**	-15.60	3892.20	134.57	< 0.0001	25.06	10050.03	362.63	< 0.0001	-14.51	3368.08	95.10	< 0.0001	-5.08	413.51	6.56	0.0209
**B**	5.96	568.23	19.65	0.0005	-	-	-	-	-	-	-	-	- 2.69	115.43	1.83	0.1948
**D**	-4.14	273.65	9.46	0.0077	-	-	-	-	-2.18	75.94	2.14	0.1625	-	-	-	-
**AB**	-	-	-	-	-	-	-	-	-	-	-	-	4.87	380.13	6.03	0.0259
**AD**	-	-	-	-	-	-	-	-	7.14	815.19	23.02	0.0002	-	-	-	-
**A^2**	-14.81	702.17	24.28	0.0002	-	-	-	-	-	-	-	-	-	-	-	-
**Residual**	-	28.92	-	-	-	27.71	-	-	-	35.42	-	-	-	63.04	-	-
**Lack of fit**	-	20.58	0.3304	0.9288	-	28.34	1.15	0.5201	-	38.60	1.79	0.3493	-	74.56	5.69	0.0890
**Pure error**	-	62.30	-	-	-	24.57	-	-	-	21.62	-	-	-	13.09	-	-

	**S.D.***	5.38	**r^2***	0.9261	**S.D.***	5.26	**r^2***	0.9527	**S.D.***	5.95	**r^2***	0.8826	**S.D.***	7.94	**r^2***	0.4741
	**Mean**	58.46	**Adj r^2***	0.9064	**Mean**	66.54	**Adj r^2***	0.9501	**Mean**	49.40	**Adj r^2***	0.8606	**Mean**	79.29	**Adj r^2***	0.3754
	**C.V. %***	9.20	**Pred r^2***	0.8686	**C.V. %***	7.91	**Pred r^2***	0.9412	**C.V. %***	12.05	**Pred r^2***	0.8107	**C.V. %***	10.01	**Pred r^2***	0.1407
	**PRESS***	771.29	**Adeq Precision***	19.1086	**PRESS***	620.50	**Adeq Precision***	30.109	**PRESS***	913.35	**Adeq Precision***	16.2665	**PRESS***	1647.75	**Adeq Precision***	5.6091

** S.D., standard deviation associated with the experimental error; Mean, dependent mean (average of all the values of the response); C.V.%, coefficient of variance of the model; PRESS, Predicted Residual Sum of Squares for the model; r^2, multiple correlation coefficient; Adj r^2, r^2 adjusted for the number of parameters in the model; Pred r^2, predicted r^2 is a measure of how the model predicts a response value; Adeq Precision, adequate precision a measure of the range in predicted response relative to its associated error.*

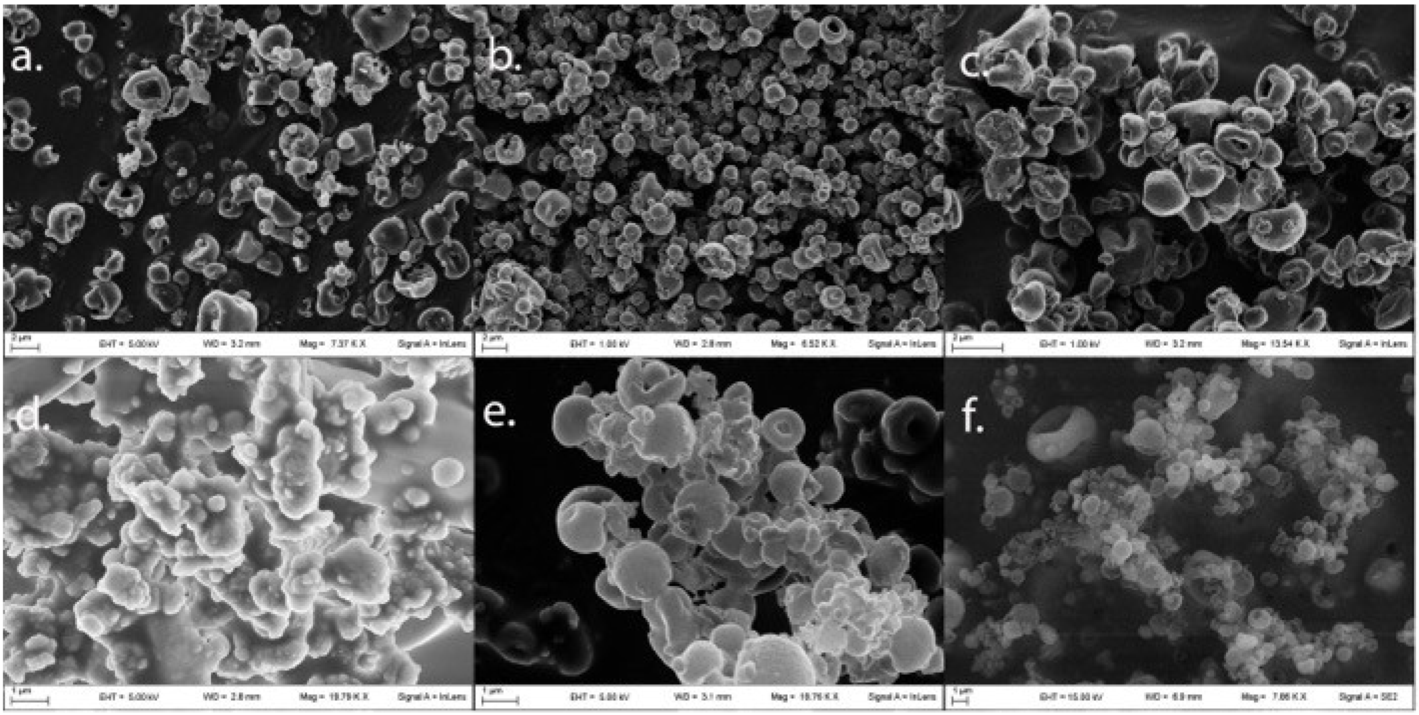

Figure S1. Morphology of representative preparations of the 20 runs of the experimental design in Table 2. In detail: a. run# 1, b. run#2, c. run#3, d. run#4, e. run#8, f. run# 10.

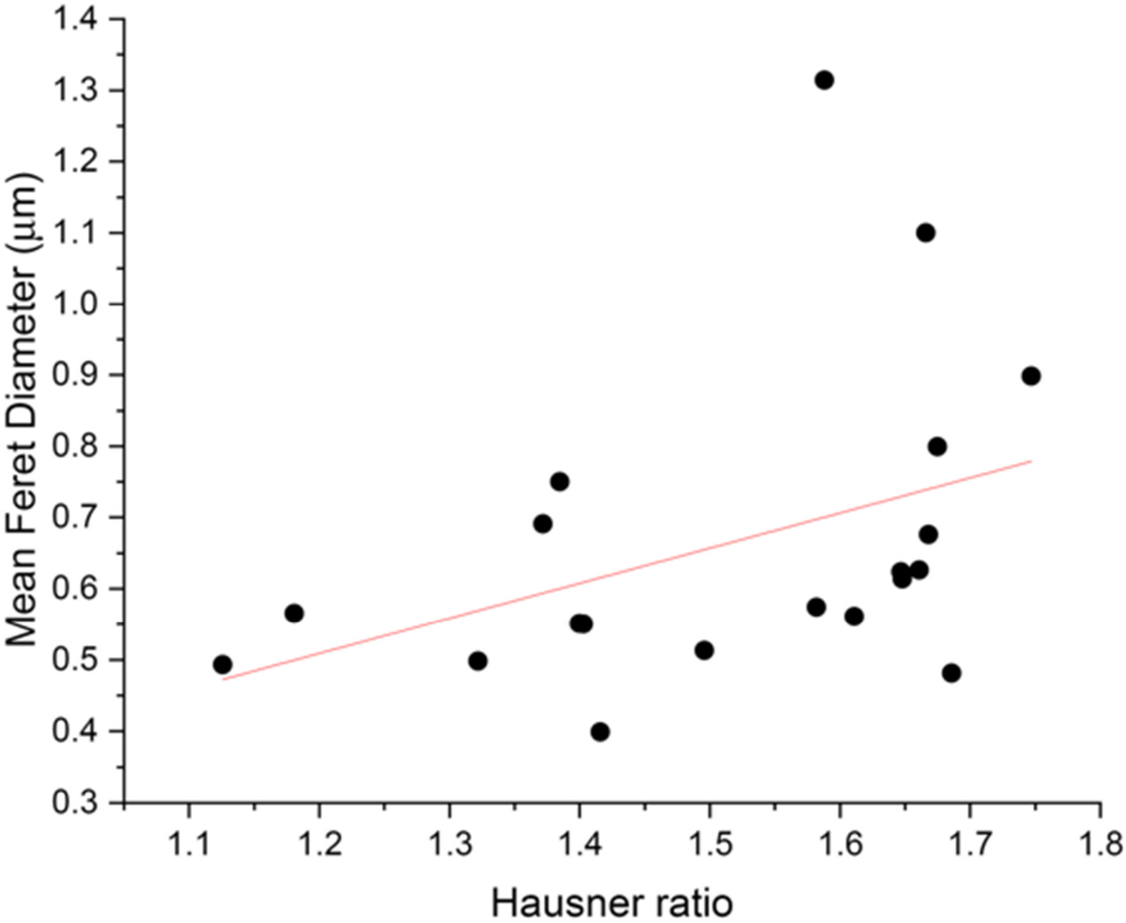

Figure S2. Correlation between theoretical flowability and particle size of the 20 runs of the experimental design in Table 2.

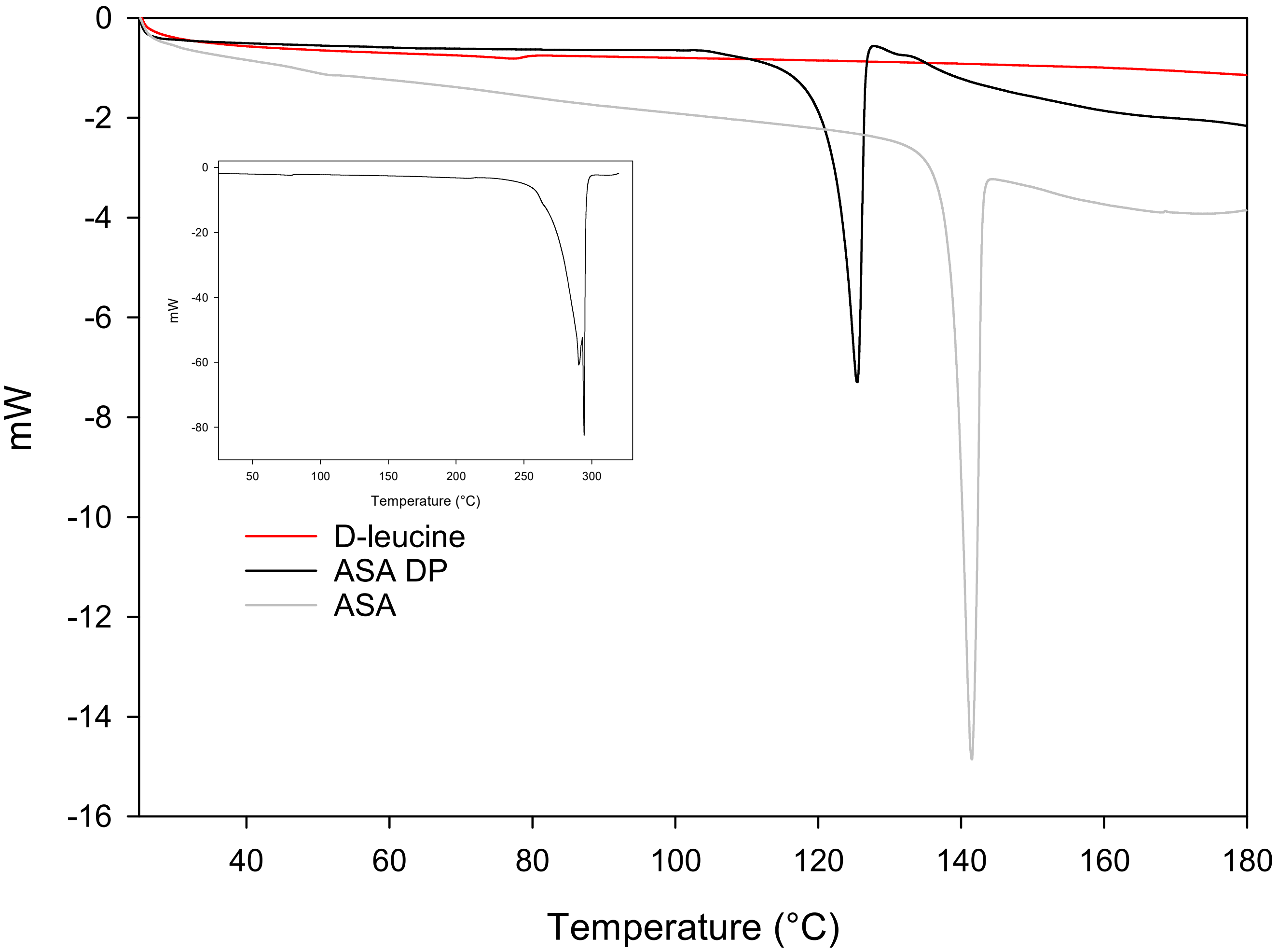

Figure S3. DSC analysis of the obtained ASA dry powder corresponding to the run# 7 of the experimental design (Table 2) (Endo down). Insert) Complete DSC profile of D-leucine showing melting and decomposition at 280-290 °C. ASA is present as a crystalline phase, note the significant downshift of the melting temperature, from 141 °C to 125 °C. This effect can be ascribed to phase mixing between ASA and D-leucine.
